# Analysis of Host Responses to *Mycobacterium tuberculosis* Antigens in a Multi-Site Study of Subjects with Different TB and HIV Infection States in Sub-Saharan Africa

**DOI:** 10.1371/journal.pone.0074080

**Published:** 2013-09-10

**Authors:** Jayne S. Sutherland, Maeve K. Lalor, Gillian F. Black, Lyn R. Ambrose, Andre G. Loxton, Novel N. Chegou, Desta Kassa, Adane Mihret, Rawleigh Howe, Harriet Mayanja-Kizza, Marie P. Gomez, Simon Donkor, Kees Franken, Willem Hanekom, Michel R. Klein, Shreemanta K. Parida, W. Henry Boom, Bonnie A. Thiel, Amelia C. Crampin, Martin Ota, Gerhard Walzl, Tom H. M. Ottenhoff, Hazel M. Dockrell, Stefan H. E. Kaufmann, and the GCGH Biomarkers for TB consortium

**Affiliations:** 1 Vaccinology Theme, Medical Research Council Unit, Fajara, The Gambia; 2 Department of Immunology and Infection, London School of Hygiene and Tropical Medicine, London, United Kingdom; 3 Department of Biomedical Sciences, Faculty of Medicine and Health Sciences, Stellenbosch University, Tygerberg, South Africa; 4 Karonga Prevention Study, Chilumba, Malawi; 5 Infectious and Non-infectious Diseases Research Directorate, Ethiopian Health & Nutrition Research Institute, Addis Ababa, Ethiopia; 6 Immunology Unit, Armauer Hansen Research Institute, Addis Ababa, Ethiopia; 7 Department of Medicine, Makerere University, Kampala, Uganda; 8 Department of Infectious Diseases, Leiden University Medical Center, Leiden, The Netherlands; 9 University of Cape Town, Cape Town, South Africa; 10 Department of Immunology, Max Planck Institute for Infection Biology, Berlin, Germany; 11 Case Western Reserve University, Cleveland, Ohio, United States of America; Fundació Institut d’Investigació en Ciències de la Salut Germans Trias i Pujol. Universitat Autònoma de Barcelona. CIBERES, Spain

## Abstract

**Background:**

Tuberculosis (TB) remains a global health threat with 9 million new cases and 1.4 million deaths per year. In order to develop a protective vaccine, we need to define the antigens expressed by *Mycobacterium tuberculosis* (Mtb), which are relevant to protective immunity in high-endemic areas.

**Methods:**

We analysed responses to 23 Mtb antigens in a total of 1247 subjects with different HIV and TB status across 5 geographically diverse sites in Africa (South Africa, The Gambia, Ethiopia, Malawi and Uganda). We used a 7-day whole blood assay followed by IFN-γ ELISA on the supernatants. Antigens included PPD, ESAT-6 and Ag85B (dominant antigens) together with novel resuscitation-promoting factors (rpf), reactivation proteins, latency (Mtb DosR regulon-encoded) antigens, starvation-induced antigens and secreted antigens.

**Results:**

There was variation between sites in responses to the antigens, presumably due to underlying genetic and environmental differences. When results from all sites were combined, HIV- subjects with active TB showed significantly lower responses compared to both TST^-^ and TST^+^ contacts to latency antigens (Rv0569, Rv1733, Rv1735, Rv1737) and the rpf Rv0867; whilst responses to ESAT-6/CFP-10 fusion protein (EC), PPD, Rv2029, TB10.3, and TB10.4 were significantly higher in TST^+^ contacts (LTBI) compared to TB and TST^-^ contacts fewer differences were seen in subjects with HIV co-infection, with responses to the mitogen PHA significantly lower in subjects with active TB compared to those with LTBI and no difference with any antigen.

**Conclusions:**

Our multi-site study design for testing novel Mtb antigens revealed promising antigens for future vaccine development. The IFN-γ ELISA is a cheap and useful tool for screening potential antigenicity in subjects with different ethnic backgrounds and across a spectrum of TB and HIV infection states. Analysis of cytokines other than IFN-γ is currently on-going to determine correlates of protection, which may be useful for vaccine efficacy trials.

## Introduction

*Mycobacterium tuberculosis* (Mtb) complex is the causative agent of tuberculosis (TB). The scientific challenges in understanding immunity to Mtb arise from the observation that, although immune responses are generated after infection, eradication of the bacteria is rare [1]. Instead, host immunity causes Mtb to adopt a clinically silent, latent state of infection in which it is highly resistant to immune attack. Once immunity becomes dysregulated the bacteria can become reactivated [1]. Considering that over 2 billion people live with latent TB infection (LTBI) [2], this population provides an enormous reservoir for potentially new cases of active TB disease.

The Mtb life-cycle can be separated into three main stages: latent (dormant), reactivating and active TB. Each stage represents differences in Mtb gene expression and therefore determining the immune response to stage-specific antigens can inform the design of new vaccine candidates [3,4]. For instance, in LTBI, the Mtb DosR regulon is induced by conditions that inhibit aerobic respiration and prevent bacillary replication and is crucial for rapid resumption of growth by involving resuscitation-promoting factors (rpf) once Mtb exits the hypoxic/anaerobic or nitric oxide-induced non-respiring state [5]. Once reactivation has occurred, the induction of a strong immune response by the host may actually provide further benefit to the bacteria [1–3]: T cell responses to TB antigens have been shown to be significantly higher in active TB than LTBI [6,7] suggesting that increased immunity may promote lung pathology and subsequently transmission [1]. Indeed, immunogenicity does not necessarily equate to protection, as illustrated by the recent failure of a novel prime-boost vaccine, MVA85A, to protect children against TB [8], despite a proven antigen-specific T cell response [9]. Thus, more information is required to understand what constitutes protective immunity to TB and in turn to inform new vaccine design strategies.

Our consortium previously reported responses to 51 DosR antigens in latently infected HIV^-^subjects from Uganda, South Africa and The Gambia with Rv1733c being the most commonly recognised antigen [10]. However, whilst similarities between sites were observed, there were also significant differences between the populations. Another study of South African subjects showed that responses to rpf were significantly higher in TB cases compared to household contacts (HHC) but values were minute compared to responses to dominant antigens such as ESAT-6/CFP-10 fusion protein (EC) [11] and comprised a mixture of uninfected and latently infected HHC. Building on these preliminary findings, the present study analysed T cell responses to 23 Mtb antigens in a total of 1247 subjects with different HIV and TB status across five geographically diverse sites in Africa (South Africa, The Gambia, Ethiopia, Malawi and Uganda).

## Methods

### Ethics statement

This study was conducted according to the principles expressed in the Declaration of Helsinki. Study protocols were approved by specific review boards at each institution (full details for each site are listed in the Table **S1**). All patients provided written informed consent for the collection of samples and subsequent analysis.

### Subjects and Study sites

The study design for GC6-74 has been previously described [12]. For this sub-study, sites included The Gambia (Medical Research Council, MRC), Ethiopia (Armauer Hansen Research Institute, AHRI), Malawi (Karonga Prevention Study, KPS), Uganda (Makerere University, MAK) and South Africa (Stellenbosch University, SUN). Subjects were considered for inclusion if they were ≥18 years of age, had no concurrent infections and were willing to undergo an HIV test. Subjects without TB were recruited from households of TB patients (MRC, SUN, and MAK) or by random community selection and from HIV care clinics (KPS, AHRI) (termed household and community controls; HCC). All subjects underwent a clinical assessment, including a chest x-ray and a screen for malaria and inter-current illnesses. Tuberculin skin tests (TST; two tuberculin units [TU], PPD RT23, SSI, Denmark) were performed in order to detect latent infection status in the subjects without active disease. Subjects with induration ≥10mm for HIV^-^ or ≥5mm for HIV^+^ subjects were classified as latently infected (TST^+^). TB cases were confirmed by sputum culture (BACTEC™, Becton-Dickinson, USA). If BACTEC was not available, culture on Lowenstein-Jensen solid media was performed (KPS, AHRI). Following informed consent, heparinised whole blood was collected for the whole blood assay set-up.

### Seven-day whole blood assay

200µl of 1:10 diluted whole blood was stimulated with each antigen in triplicate as previously described [10]. Each site used the same batch of quality controlled antigens, controls and reagents. The antigens used were generated by our consortium and immunogenicity determined previously [10,13] (Table **S2**). Following 7 days incubation at 37^o^C, 5% CO_2_, supernatants were harvested and stored at -20^o^C prior to analysis by IFN-γ ELISA. Antigens were evaluated at a final concentration of 10µg/ml except for the peptide pools, Rv2659c and Rv2660, which were used at 1µg/mL final concentration per peptide and Mtb PPD, which was used at 5µg/mL final concentration. Controls included unstimulated (negative control) and the polyclonal stimulator, phytohaemagglutinin (PHA 5µg/mL; positive control) (Sigma, USA). Antigens were produced at SSI (Denmark) or LUMC (The Netherlands).

### IFN-γ ELISA

Supernatants were analysed by IFN-γ ELISA as previously described [10,14]. Briefly, plates were coated overnight with mouse anti-human IFN-γ monoclonal antibody (2µg/ml; Becton-Dickinson, USA) at 4^o^C. Following washing with PBS-Tween 20, wells were blocked using PBS/FCS (Sigma, USA) for 2 hours. Samples, controls and standards were then added and the plate incubated overnight at 37^o^C. After washing, biotinylated anti-rabbit detection antibody (1µg/mL; Becton-Dickinson, USA) was added, plates incubated for 45min. at RT and a final colorimetric step performed by addition of avidin-peroxidase followed by OPD Fast (Both from Sigma, USA). The reaction was stopped with 2M H_2_SO_4_ and the plates were read at 492nm with a four-parameter curve-fit. Each site used an aliquot of the same positive PHA-stimulated whole blood culture as a positive control. Additionally, the same batches of antibodies and standards were used in all the sites, using a standardised protocol.

### Data analysis

We assumed non-parametric distribution of samples. All antigen-stimulated wells were adjusted for non-specific responses by background subtraction (media alone). IFN-γ levels below 16pg/mL were considered as non-responses and adjusted to ‘1’. A Kruskal-Wallis test followed by Dunn’s post-test comparison was used for determining differences between TB cases and TST^+^ and TST^-^ HHC. A Mann–Whitney U-test was used for analysis of TB cases and TST^+^ controls at each site. Significance was defined as p-value ≤0.035 to adjust for false-discovery rates (FDR) with multiple comparison testing. For HIV+ subjects, analysis of CD4 counts within and between sites was performed using Kruskal-Wallis test and Dunn’s post-test comparison. Due to differences between sites in regards to CD4 counts, HIV+ subjects were analysed both with unadjusted and adjusted CD4 counts using linear regression. Logistic regression and receiver-operator curve (ROC) analyses were performed to determine which parameters best discriminate between TB and TST^+^ controls. Analyses were performed using Graphpad Prism 6 (Software MacKiev, USA) and SPSSv20 (IBM, USA).

## Results

### Subject demographics

The majority of subjects in this study were HIV^-^ with a total of 262 HIV^-^TB^+^, 454 HIV^-^TST^+^ (HCC) and 204 HIV^-^TST^-^ HCC from all sites combined (Table **1** depicts numbers from individual sites). No HIV^+^ subjects were analysed from MRC due to the low rate of HIV infection in The Gambia. For HIV-infected subjects, a total of 77 TB, 87 TST^+^ HCC and 163 TST^-^ HCC were analysed (Table **1**). There were significant differences between and within sites in regards to age and sex of the subjects so these were adjusted for accordingly in the statistical analyses where possible. A total of 71 TB index cases from MRC were also confirmed to be infected with either Mtb sensu stricto (n=39) or *M* *. africanum* (Maf; n=32); a strain only present in West Africa [15]. No significant difference in response to any of the antigens was seen in Mtb or Maf infected subjects (data not shown) and therefore this was not adjusted for in our analyses. HIV+ subjects had significant differences in CD4 counts within and between sites (Table **1**). Whilst no difference was seen for TB cases or TST+ HCC, TST- HCC were significantly different between sites particularly for SUN (p<0.05 compared to KPS and p<0.001 compared to both AHRI and MAK). In addition, TB cases had significantly lower CD4 counts compared to TST+ contacts at AHRI, KPS and MAK (p=0.01, p=0.001 and p=0.0001 respectively) but not at SUN. As such, results are presented using unadjusted values, but also with adjustment for differences in CD4 counts.

**Table 1:** Participant information.

	**HIV^+^**			**HIV^-^**			
**Site/TB status**	**TB**	**TST^+^**	**TST^-^**	**TB**	**TST^+^**	**TST^-^**	**Total**
**SUN**	23	15	47	65	175	27	**352**
Age	34[28–40]	39[30–44]	39[31–47]	28[22–40]	26[21–33]	24[19–37]	
% males	57	56	43	40	40	37	
CD4 cells/μL	191	184	153				
**MRC**	n/a	n/a	n/a	77	120	124	**321**
Age	n/a	n/a	n/a	28[23–39]	25[20–34]	24[18–31]	
% males	n/a	n/a	n/a	85	50	56	
**AHRI**	19	18	15	31	28	11	**122**
Age	35[27–45]	30[25–35]	32[24–36]	23[21–28]	31[20–45]	23[20–30]	
% males	41	14	20	65	36	27	
CD4 cells/μL	165	380	354				
**KPS**	21	39	78	32	34	18	**222**
Age	32[27–38]	39[31–48]	39[31–45]	49[29–58]	39[32–50]	34[27–52]	
% males	52	33	36	56	51	44	
CD4 cells/μL	46	328	209				
**MAK**	14	15	23	57	97	24	**230**
Age	28[25–39]	35[31–40]	30[27–39]	25[22–30]	26[19–35]	22[20–26]	
% males	43	13	30	49	43	38	
CD4 cells/μL	135	487	403				
**Total (n=)**	**77**	**87**	**163**	**262**	**454**	**204**	**1247**

HIV^+^ = human immunodeficiency virus-positive; HIV^–^ = human immunodeficiency virus-negative; TB = active tuberculosis; TST^+^ = tuberculin skin test-positive control; TST^–^ = tuberculin skin test-negative control; SUN = Stellenbosch University, South Africa; MRC = Medical Research Council, The Gambia; AHRI = Armauer Hansen Research Institute, Ethiopia; KPS = Karonga Prevention Study, Malawi; MAK = Makerere University, Uganda; Age = median[Interquartile range]. Total = total subjects per site per TB/HIV group. CD4 counts indicated for HIV-positive subjects only.

### IFN-γ responses to 23 antigens following a 7-day whole blood assay

**Figure 1:**
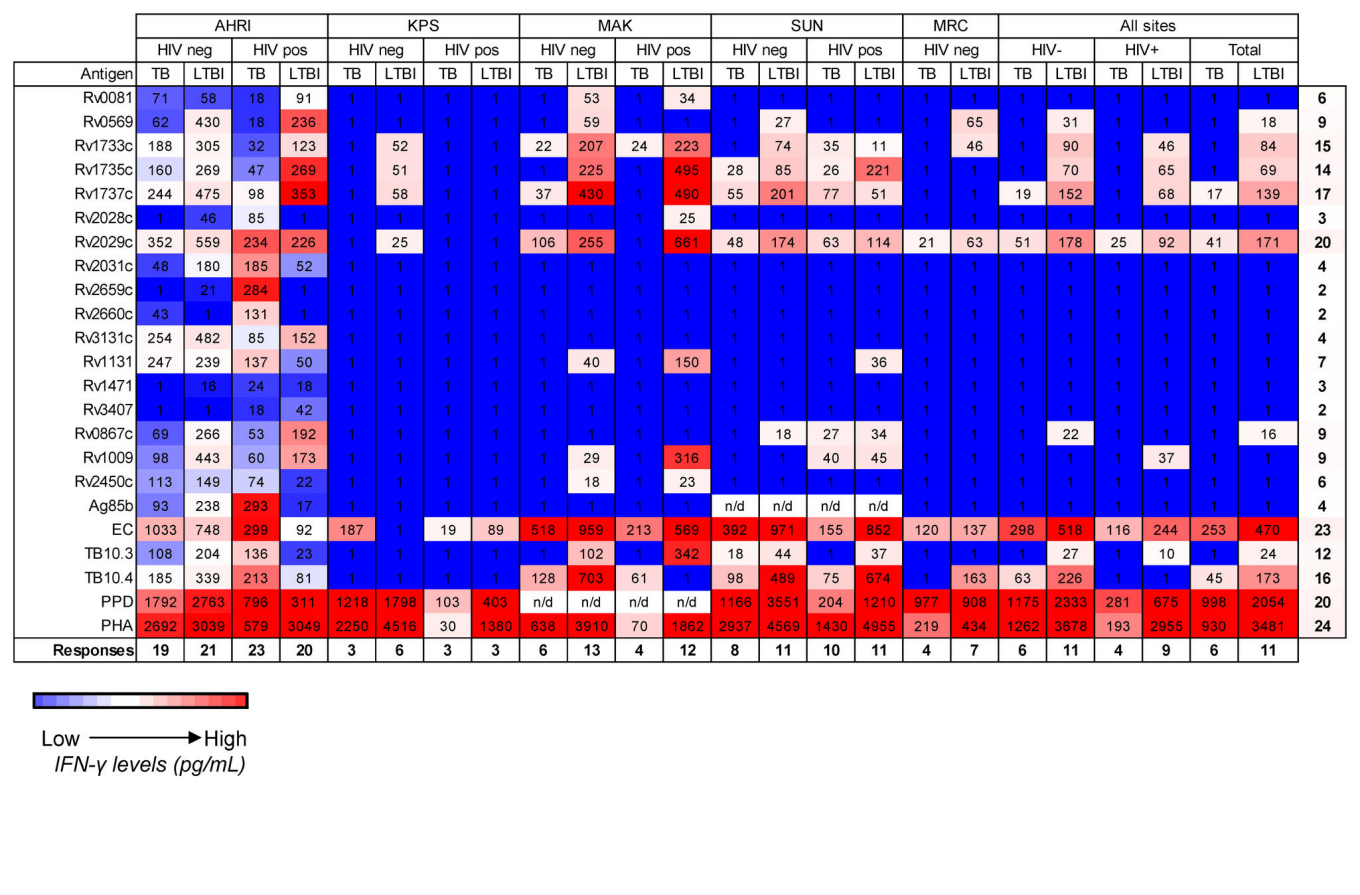
Heat map of IFN-γ responses to secreted, latent, and reactivation Mtb antigens stratified according to HIV status, TB status and location. Median levels of IFN-γ are shown (pg/mL). Red indicates relatively high levels of IFN-γ and blue indicates relatively low levels. HIV^+^ = human immunodeficiency virus positive; HIV^-^ = human immunodeficiency virus negative; TB = active tuberculosis; LTBI = latently TB infected; n/d = not done; SUN = Stellenbosch University, South Africa; MRC = Medical Research Council, The Gambia; AHRI = Armauer Hansen Research Institute, Ethiopia; KPS = Karonga Prevention Study, Malawi; MAK = Makerere University, Uganda. The right column indicates the number of groups (out of a possible 24) who responded to a particular antigen. PPD was not used at MAK nor was Ag85b used at SUN, so the maximum number for these is 20.

Figure **1** shows a heat-map of responses from all sites with subjects separated based on TB and HIV status. The highest responses were to PHA, PPD, EC, TB10.4 and Rv2029c (Figure **1**, red). The heat map illustrates the differences seen between sites, with subjects from AHRI showing responses (median) to the majority of antigens used regardless of HIV or TB status (Figure **1**; AHRI); whilst KPS subjects responded to, at most, four antigens other than PPD and PHA (HIV^-^LTBI: Rv1733, Rv1735, Rv1737 and Rv2029) and only ESAT-6/CFP10 (EC) for the other 3 groups (Figure **1**; KPS). The most consistent differences were seen to EC regardless of site, TB or HIV status. However the direction differed between sites with higher EC responses in TB patients compared to LTBI for both HIV^-^ and HIV^+^ subjects from AHRI and HIV^-^ subjects from KPS but lower responses to LTBI from all other sites regardless of HIV status. After PHA, PPD and EC, the antigens that induced the greatest number of responses from each group were Rv2029c (20 out of a possible 24 HIV/TB groups) followed by Rv1737c (17/24) and TB10.4 (16/24) (Figure **1**).

### Control Stimulants

**Figure 2:**
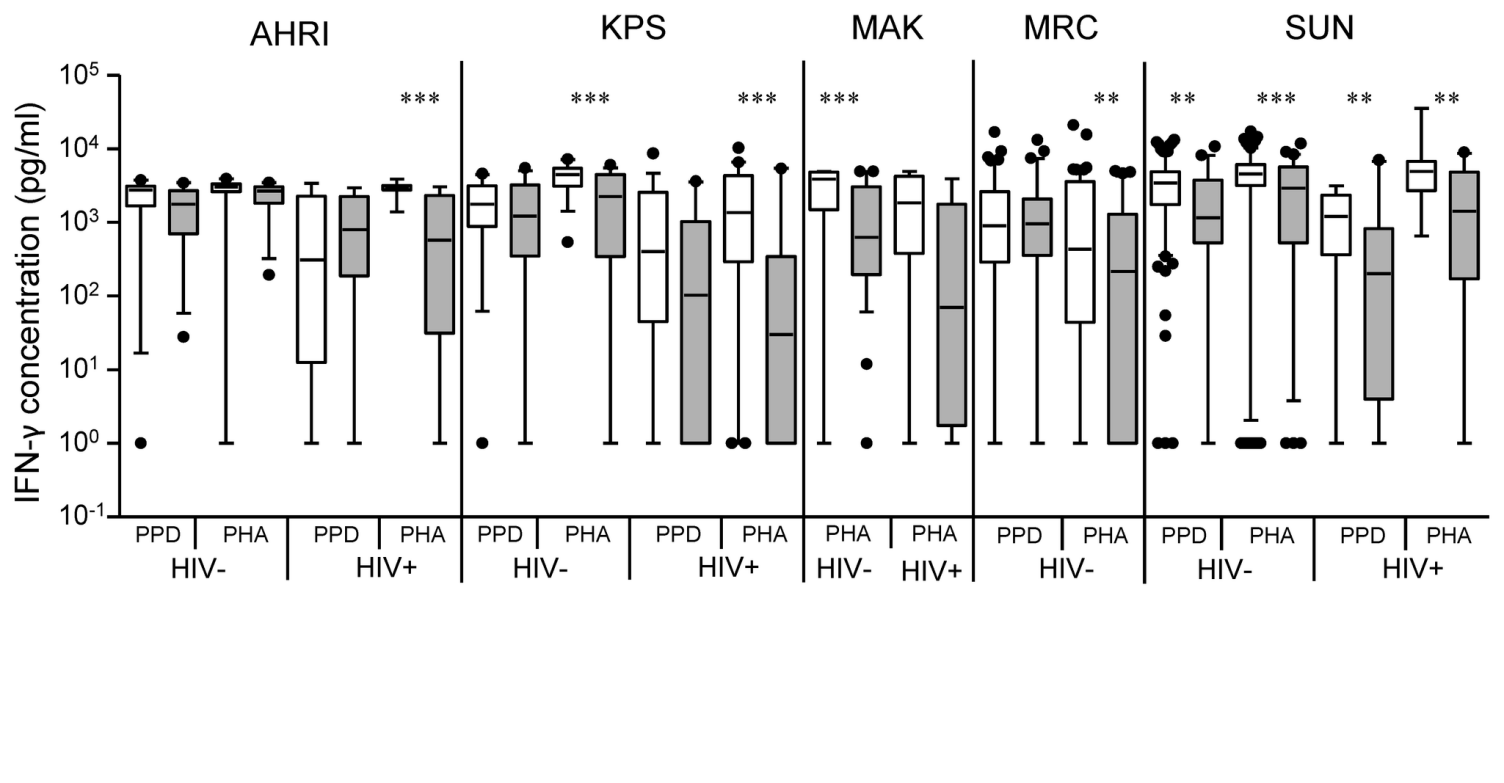
IFN-γ secretion in response to secreted, latent, and reactivation Mtb antigens in active TB and LTBI subjects, from five African sites. IFN-γ ELISA was performed on supernatants collected after 7-day antigen stimulation of diluted blood from TB cases (grey) and TST^+^ (LTBI) controls (white) from five sites in Africa. Line indicates median, whiskers indicate 5–95% range and dots indicate outliers. Data were analysed by Mann-Whitney U-test within sites for HIV^–^ and/or HIV^+^ subjects. Significant differences are indicated: *=p<0.05; **=p<0.01; ***=P<0.001.

Control antigens used in this study included PPD and the mitogen PHA as a polyclonal stimulator. PPD cross-reacts with both environmental Mycobacteria and BCG vaccine and therefore is not specific for TB in the endemic countries analysed in this study. Indeed, few differences were seen in responses to PPD between TST^+^ controls (LTBI) and active TB subjects (Figure **2**). SUN was the only site to see a difference between the groups in response to PPD: both HIV^-^ (p<0.01) and HIV^+^ (p<0.01) subjects with responses in active TB disease being significantly lower than in LTBI. Responses to the positive control PHA were significantly lower in subjects with active TB disease compared to LTBI for all sites studied regardless of HIV status. Intriguingly, these responses were often lower than for PPD (Figure **2**). Whilst there was considerable variation in PHA responses within and between sites, we did not adjust for this since responses to PPD, which reflect TB relevant immune responses, were not significantly different.

### Secreted antigens

**Figure 3:**
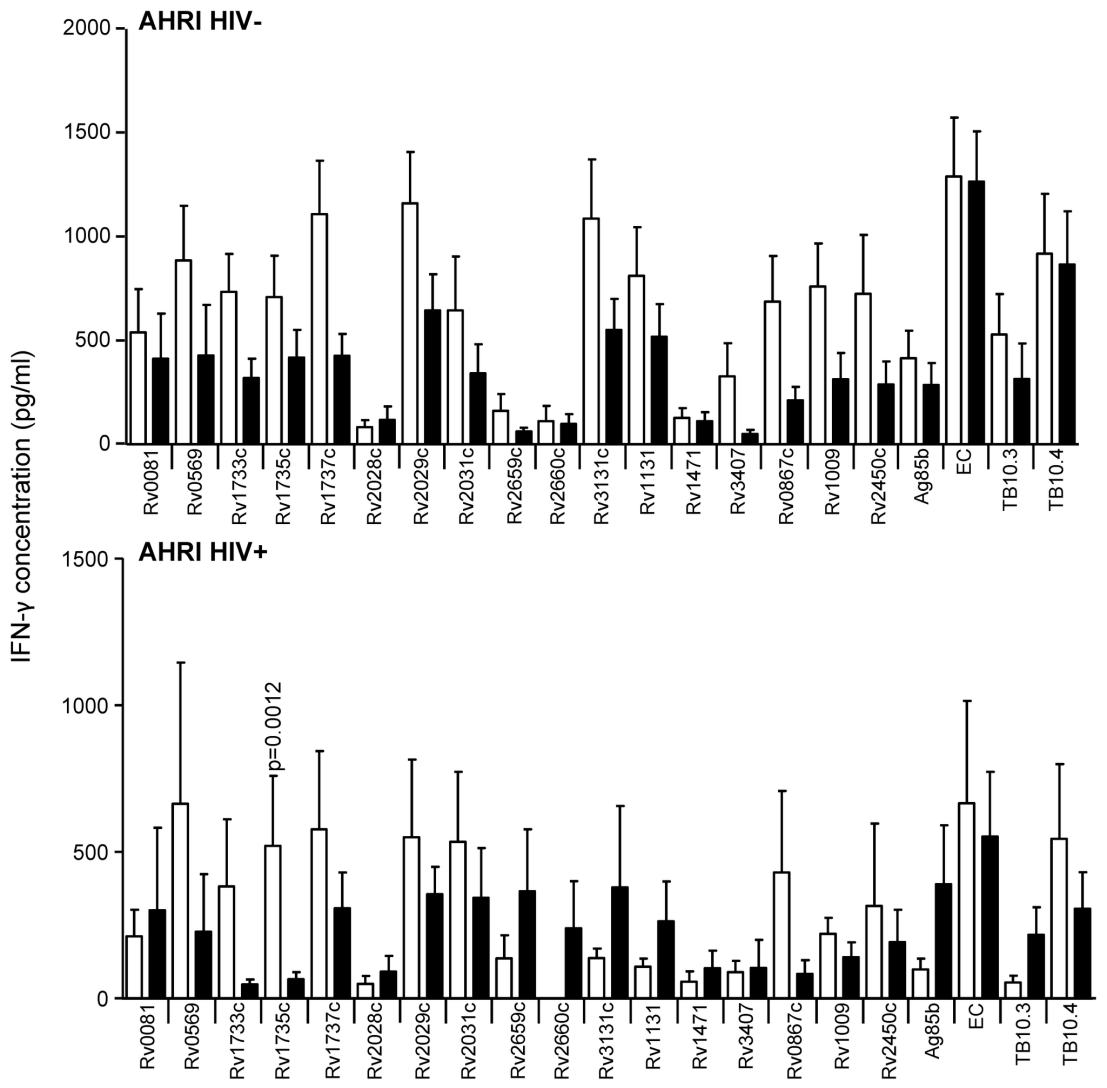
IFN-γ secretion in response to secreted, latent, and reactivation Mtb antigens in active TB and LTBI subjects, from Ethiopia. IFN-γ ELISA was performed on supernatants collected after 7 day antigen stimulation of diluted blood from TB cases (black) and TST^+^ (LTBI) controls (white) from AHRI (Ethiopia). Line indicates median, whiskers indicate 5–95% range and dots indicate outliers. Data were analysed by Mann-Whitney U-test for HIV^–^ and/or HIV^+^ subjects. Significant differences are indicated.

**Figure 4:**
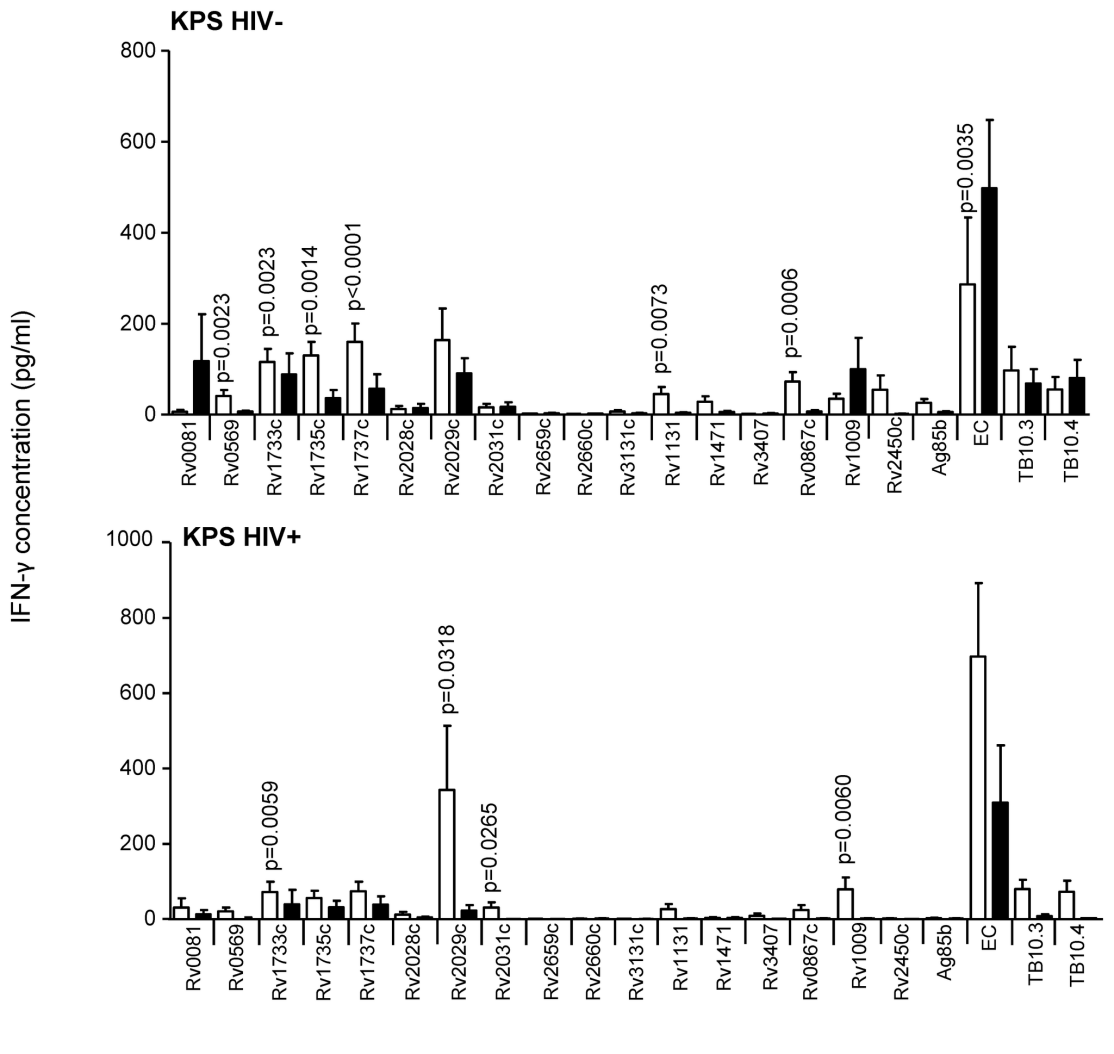
IFN-γ secretion in response to secreted, latent, and reactivation Mtb antigens in active TB and LTBI subjects, from Malawi. IFN-γ ELISA was performed on supernatants collected after 7-day antigen stimulation of diluted blood from TB cases (black) and TST+ (LTBI) controls (white) from KPS (Malawi). Line indicates median, whiskers indicate 5–95% range and dots indicate outliers. Data were analysed by Mann-Whitney U-test for HIV^–^ and/or HIV^+^ subjects. Significant differences are indicated.

**Figure 5:**
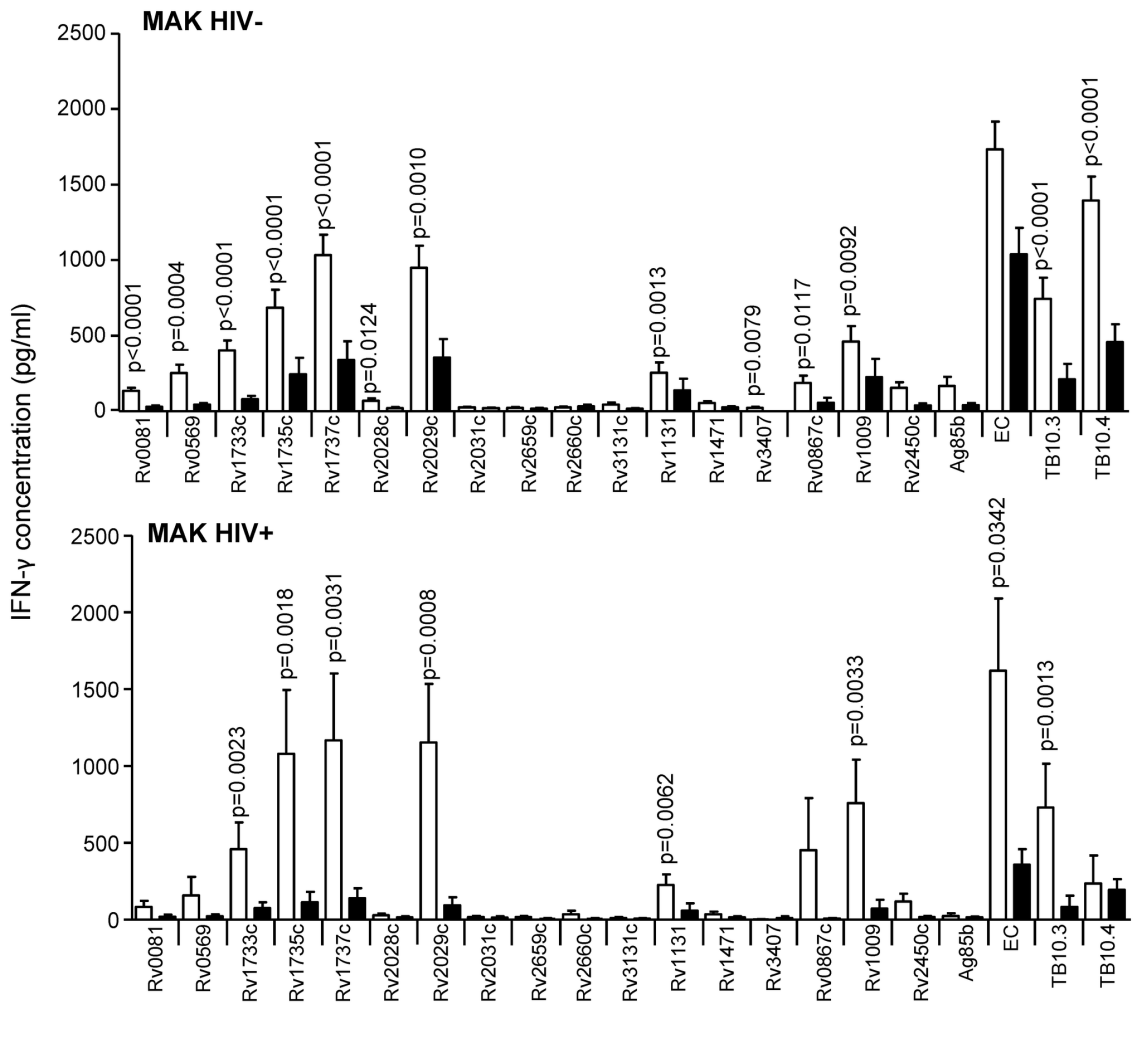
IFN-γ secretion in response to secreted, latent, and reactivation Mtb antigens in active TB and LTBI subjects, from Uganda. IFN-γ ELISA was performed on supernatants collected after 7-day antigen stimulation of diluted blood from TB cases (black) and TST^+^ (LTBI) controls (white) from MAK (Uganda). Line indicates median, whiskers indicate 5–95% range and dots indicate outliers. Data were analysed by Mann-Whitney U-test for HIV^–^ and/or HIV^+^ subjects. Significant differences are indicated.

**Figure 6:**
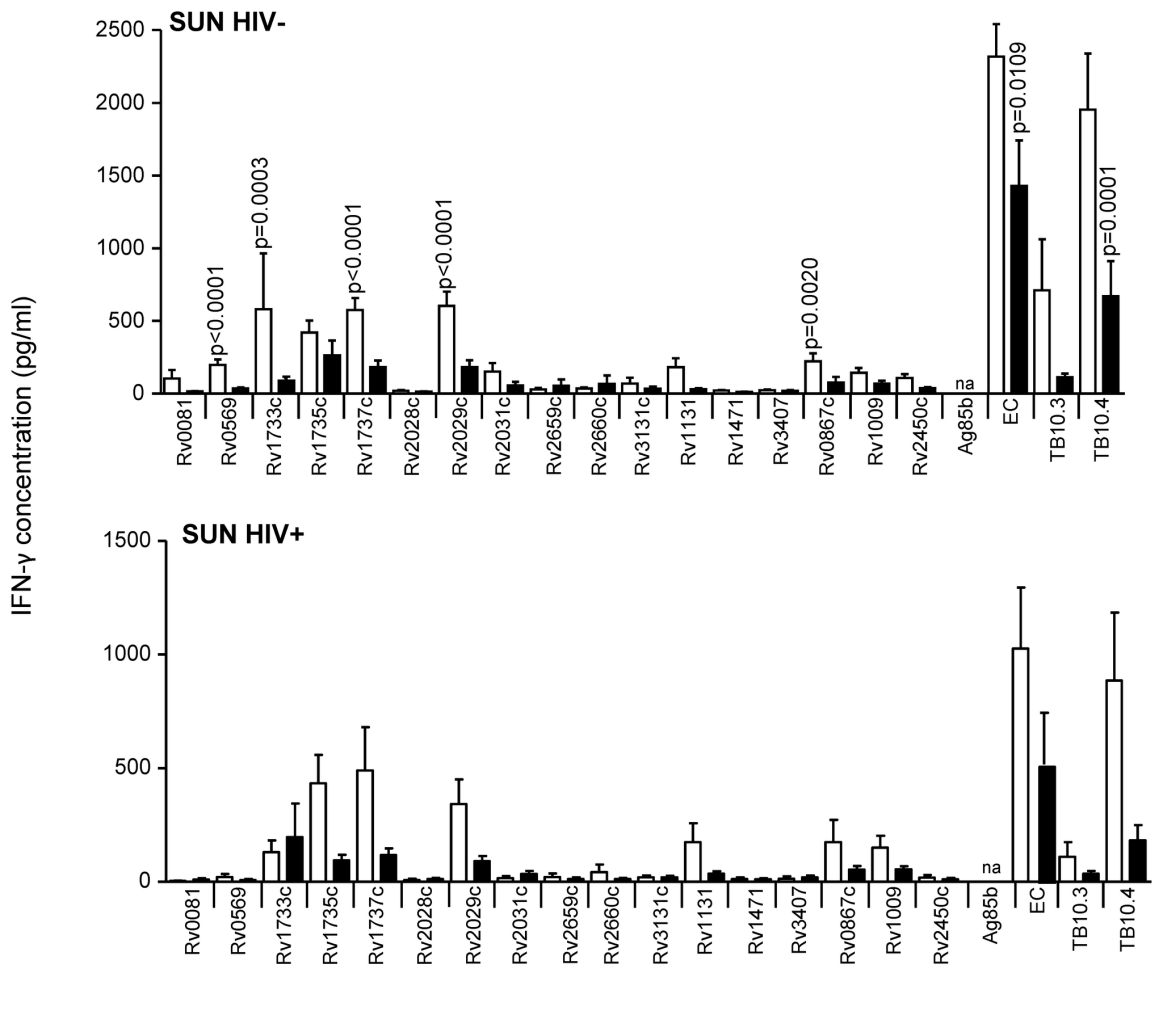
IFN-γ secretion in response to secreted, latent, and reactivation Mtb antigens in active TB and LTBI subjects, from South Africa. IFN-γ ELISA was performed on supernatants collected after 7-day antigen stimulation of diluted blood from TB cases (black) and TST^+^ (LTBI) controls (white) from SUN (South Africa). Line indicates median, whiskers indicate 5–95% range and dots indicate outliers. Data were analysed by Mann-Whitney U-test for HIV^–^ and/or HIV^+^ subjects. Significant differences are indicated. n/d = antigen not analysed.

**Figure 7:**
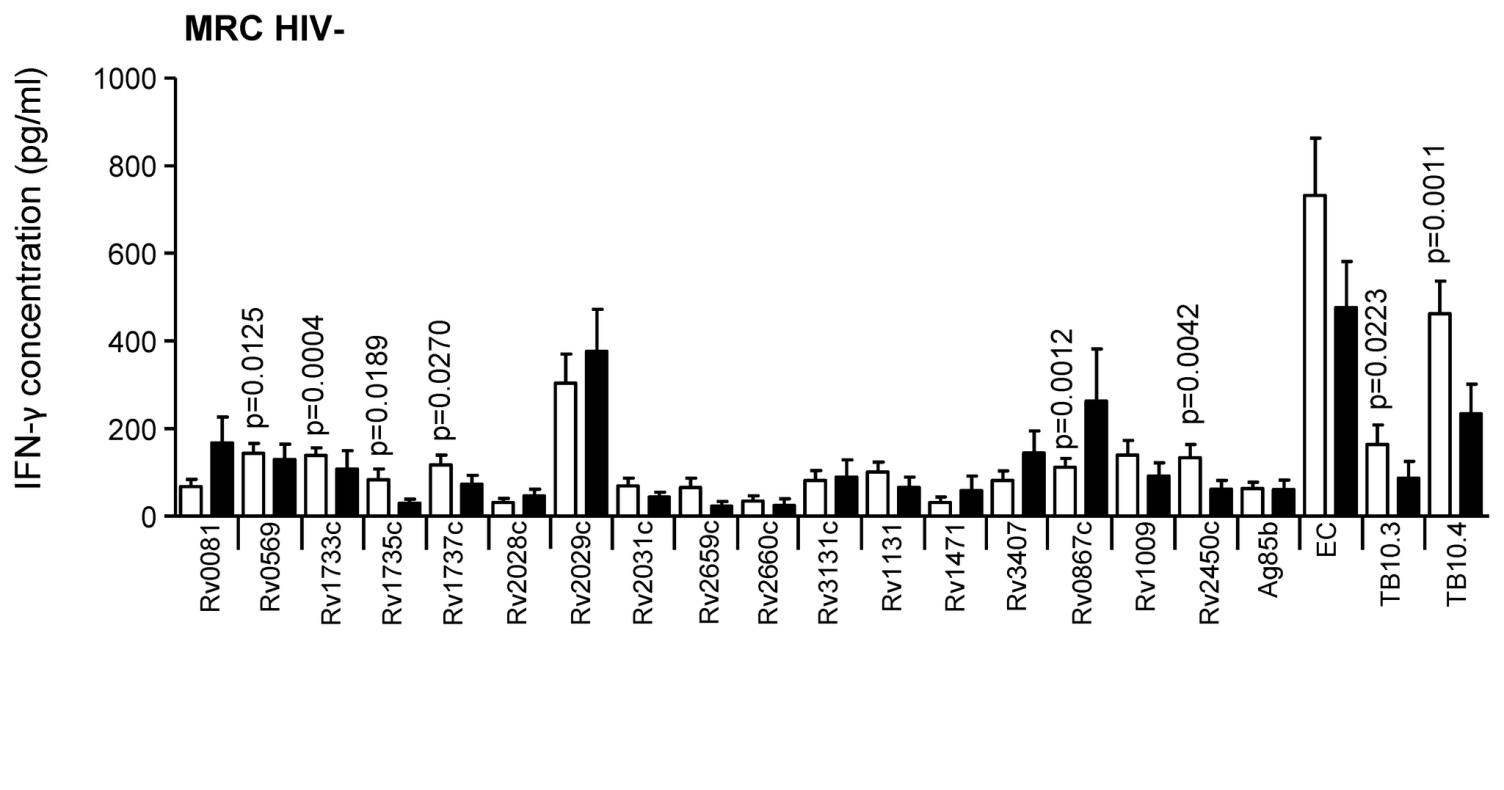
IFN-γ secretion in response to secreted, latent, and reactivation Mtb antigens in active TB and LTBI subjects, from The Gambia. IFN-γ ELISA was performed on supernatants collected after 7-day antigen stimulation of diluted blood from TB cases (black) and TST^+^ (LTBI) controls (white) from MRC (The Gambia). Line indicates median, whiskers indicate 5–95% range and dots indicate outliers. Data were analysed by Mann-Whitney U-test for HIV^–^ subjects. Significant differences are indicated.

The secreted Mtb antigens used in this study were EC, Rv0288 (TB10.4), Rv1886 (Ag85b) and Rv3019 (TB10.3) (Table **S2**). There were no significant differences between TB and LTBI in response to these antigens with subjects from AHRI (HIV^-^ and HIV^+^; Figure **3**). HIV^-^ TB cases from KPS had a significantly higher response to EC than LTBI (p=0.0035; Figure **4A**), but no difference in HIV+ subjects after adjusting for FDR (Figure **4B**). LTBI subjects from Uganda (MAK) had significantly higher IFN-γ levels compared to TB following TB10.3 and TB10.4 stimulation (p<0.0001 for both) in HIV^-^ subjects (Figure **5A**) and following EC (p=0.0342) and TB10.3 (0=0.0013) stimulation in HIV^+^ subjects (Figure **5B**). However, when CD4 counts were adjusted, only TB10.3 responses were significantly different (p=0.026). Ag85b responses were not assessed in subjects from SUN, however, responses to EC and TB10.4 were both significantly higher in HIV^-^ LTBI compared to HIV^-^ TB (p=0.0109 and p=0.0001 respectively; Figure **6A**). Similarly, HIV^+^ LTBI subjects from SUN had higher levels of IFN-γ in response to EC and TB10.4 than HIV^+^ TB after adjusting for CD4 counts (p=0.003 and p<0.0001 respectively; Figure **6B**). Due to low levels of HIV infection in The Gambia, data from MRC is presented for HIV^-^ subjects only (Figure **7**). Similar to AHRI and MAK, LTBI subjects from MRC had higher responses to secreted antigens than those with active TB with a significant difference in IFN-γ production following stimulation with TB10.3 (p=0.0223) and TB10.4 (p=0.0001) and a higher (but not significant) response to EC (Figure **7**).

### Reactivation antigens

The reactivation antigens used in this study were Rv1131, Rv1471, Rv3407, Rv0867c, Rv1009 and Rv2450c (Table **S2**); the latter three are so-called rpf proteins. Principally, responses to these antigens were lower than for the secreted antigens except for HIV^-^ subjects from AHRI but, despite higher levels in LTBI, there were no significant differences seen between the groups for any antigen in HIV^-^ or HIV^+^ subjects from AHRI (Figure **3**). HIV^-^ LTBI from KPS showed significantly higher responses than HIV-TB to Rv1131 and Rv0867c (p=0.0073 and p=0.006 respectively; Figure **4A**). HIV^+^ subjects from KPS showed low responses to all of the reactivation antigens except Rv1009, which was significantly higher in LTBI than TB (p=0.0060; Figure **4B**). After adjusting for CD4 levels, Rv1131 was also significantly higher in LTBI compared to TB (p<0.035). HIV^-^ LTBI subjects from MAK revealed significantly higher responses to all reactivation antigens compared to HIV^-^ TB except Rv1471 and Rv2450 (Figure **5A**). HIV^+^ LTBI subjects from MAK had significantly higher levels of IFN-γ following stimulation with Rv1131 and Rv1009 compared to TB (p<0.0062 and p<0.0033 respectively; Figure **5B**) but only for Rv1009 after adjusting for CD4 counts (p=0.013). HIV^-^ subjects from SUN generated low responses to the reactivation antigens (Figure **6A**), although these were significantly higher in HIV^-^ TB compared to LTBI in response to Rv0867c (p=0.0020; Figure **6A**). HIV^+^ subjects from SUN did not respond to any reactivation antigen (Figure **6B**) even after adjusting for CD4 counts. While all other sites had higher levels of response to Rv0867c from LTBI compared to active TB, this was the reverse for HIV- subjects from MRC (p=0.012; Figure **7**). Responses to Rv2450c followed the same pattern as for the other sites with levels of IFN-γ significantly higher in LTBI than TB subjects from MRC (p=0.0042; Figure **7**).

### Latency antigens

Latency (i.e. Mtb DosR regulon-encoded) antigens included in this study were Rv0081, Rv0569, Rv1733c, Rv1735c, Rv1737c, Rv2028c, Rv2029c, Rv2031c, and Rv3131c, next to the two starvation-induced antigens Rv2659c, Rv2660c. Whilst HIV^-^ subjects from AHRI showed no significant difference between LTBI and TB to any of these antigens (although LTBI were generally higher than TB), HIV^+^ LTBI had a significantly higher response to Rv1735c than HIV^+^ TB (p=0.0012; Figure **3B**); although this was lost after adjusting for CD4 counts. HIV^-^ LTBI from KPS generated significantly higher responses to Rv0569, Rv1733c, Rv1735c and Rv1737c than HIV^-^ TB (p=0.0023, p=0.0023, p=0.0014 and p<0.0001 respectively; Figure **4A**). Additionally, HIV^+^ LTBI from KPS revealed significantly higher responses to Rv1733 and Rv2029c and Rv2031c than HIV^+^ TB (p=0.0059, p=.0318 and p=0.0265 respectively; Figure **4B**); but again no difference after adjusting for CD4 counts. HIV^-^ LTBI from MAK had significantly higher levels of IFN-γ than HIV^-^ TB following stimulation with all the latency antigens used in this study (Figure **5A**). HIV^+^ LTBI also had higher responses than HIV^+^ TB to most of the latency antigens with significant differences found for Rv1733c, Rv1735c, Rv1737c and Rv2029c (p=0.0023, p=0.0018, p=0.0031 and p=0.0008 respectively; Figure **5B**). After adjusting for CD4 counts, Rv1737c and Rv2029c remained significantly different between LTBI and TB (p=0.011 and p=0.003 respectively). HIV^-^ LTBI subjects from SUN had significantly higher levels of IFN-γ compared to HIV^-^ TB subjects in response to Rv0569, Rv1733c, Rv1737c and Rv2029c (Figure **6A**). Again, HIV^+^ subjects from SUN showed no differences in response to any of the latency antigens between LTBI and TB (Figure **6B**). Whilst MRC subjects generated lower responses to most of the latency antigens than HIV^-^ subjects from AHRI, MAK and SUN, they were significantly higher in LTBI compared to active TB subjects in response to Rv0569, Rv1733c, Rv1735c and Rv1737c.

### Comparison of TST^-^ (Mtb-uninfected), TST^+^ (LTBI) and active TB groups

**Figure 8:**
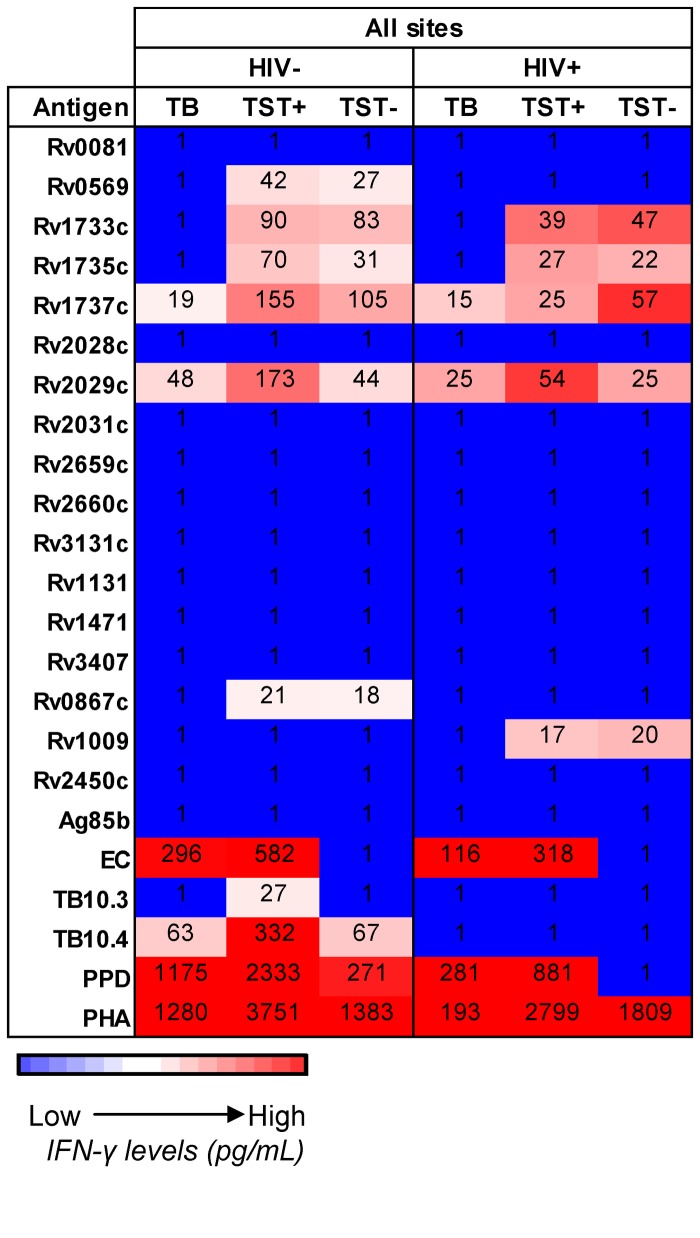
Heat map of combined responses from all sites for TST^–^, TST^+^ (LTBI) and active TB with or without HIV infection in response to secreted, latent, and reactivation Mtb antigens. Median levels of IFN-γ are shown (pg/mL). Red indicates relatively high levels of IFN-γ and blue indicates relatively low levels. HIV^+^ = human immunodeficiency virus positive; HIV^-^ = human immunodeficiency virus-negative; TB = active tuberculosis; LTBI = latently TB infected; SUN = Stellenbosch University, South Africa; MRC = Medical Research Council, The Gambia; AHRI = Armauer Hansen Research Institute, Ethiopia; KPS = Karonga Prevention Study, Malawi; MAK = Makerere University, Uganda.

We analysed HIV^-^ and HIV^+^ subjects from all sites based on their TB status (Mtb-uninfected (TST^-^), latent infection (TST^+^) or active disease (TB)). For HIV^-^ subjects, active TB subjects had significantly lower levels of IFN-γ compared to both TST^-^ and TST^+^ controls in response to latency antigens Rv0569, Rv1733, Rv1735, Rv1737 and Rv0867 (p<0.0001 for all; Figure **8**). Responses to EC and PPD were significantly higher in HIV^-^ TST^+^ (LTBI) compared to HIV^-^ TB subjects (p=0.016 and p<0.0001 respectively) who were in turn, significantly higher than HIV^-^ TST^-^ subjects (p<0.0001 for both; Figure **8**). Responses to Rv2029, TB10.3, TB10.4 and PHA were significantly higher in HIV^-^ TST^+^ compared to both HIV^-^ TB and HIV^-^ TST^-^ (who were comparable; p<0.0001 for all; Figure **8**). Fewer differences were seen with HIV^+^ subjects: the only significant difference between TB and TST^+^ subjects was in response to PHA with a median of 193 pg/mL for HIV^+^ TB^+^ compared to 2799 pg/mL for HIV^+^ TST^+^ (p<0.0001; Figure **8**). Similar to the HIV^-^ subjects, we observed a hierarchical response to EC and PPD with LTBI higher than TB (but not significantly) and both significantly higher than HIV^+^ TST^-^ (p<0.0001 for all; Figure **8**). When ROC analysis was performed, no single antigen response could discriminate between active TB disease and latent infection with >65% correct classification regardless of HIV status (data not shown). 

## Discussion

Discovery of new antigens that could provide protection against primary or reactivation TB disease is essential for development of next-generation vaccines. The fact that BCG remains the only licensed TB vaccine for over 90 years shows how difficult this accomplishment is. A recent trial using Mtb Antigen 85A as a boost to BCG in infants showed no increase in protection against development of TB, despite strong immunogenicity in Phase I/IIa trials [8]. Thus we also need to determine the exact correlates of protection to determine which antigens to incorporate in vaccine design strategies and for determining vaccine efficacy without the requirement for extremely large and expensive cohort studies. The Bill & Melinda Gates Foundation, Grand Challenges (GC) in Global Health, Biomarkers for TB: GC6-74 aims to determine biomarkers for protective immunity in the context of HIV/AIDS in Africa (http://www.biomarkers-for-tb.net/consortium). As one objective, we analysed responses from 1247 subjects to 23 Mtb antigens to elucidate the influence of TB status (latent infection, Mtb-uninfected, active TB disease), HIV status and geographical location on these responses.

The antigens analysed included PPD, ESAT-6 and Ag85B together with novel rpf, reactivation proteins, latency (Mtb DosR regulon-encoded) antigens, starvation-induced antigens and secreted antigens tentatively characterized for T-cell responses in HIV^-^ HHC in a previous pilot study of 86 antigens [10]. Antigens used in current diagnostic/vaccination strategies (TB10.4, PPD and ESAT-6/CFP-10) generated dominant responses from all sites but very few differences between active TB disease and LTBI. The next highest responses were seen to Rv2029c followed by Rv1733, Rv1735 and Rv1737. These are all dormancy-associated antigens, essential for the survival of Mtb during persistence *in vivo* and Rv1733, 1735 and 1737 were previously shown to induce dominant immunogenic responses in a small cohort of LTBI subjects from The Gambia, South Africa and Uganda [10]. Rv2029c is probable phosphofructokinase (pfkB) and is one of the most important enzymes of glycolysis [16]. A recent study suggests that glycolysis leads to accumulation of glucose-derived toxic metabolites that limits long-term survival of Mtb under hypoxic conditions [16] and is exacerbated when the glycolytic pathway is disrupted at the PKF step. Whilst HIV^+^ and HIV^-^ subjects from Uganda and HIV^+^ subjects from Malawi showed significant differences between LTBI and TB to Rv2029c, no differences were observed at the other three sites. This pattern was similar for the majority of antigens used in this study: whilst there were similarities between sites, there were also many differences. Notably, these included no differences between TB and LTBI in subjects from Ethiopia for any antigen, and much lower responses to all antigens in subjects from Malawi. Note that these differences between sites are unlikely due to differences in Mtb strain since Gambian subjects infected with Mtb *sensu stricto* or *M* *. africanum* showed no differences in response to any of the antigens (data not shown). There was a large variation in CD4 counts for HIV+ subjects within and between sites, which will clearly affect levels of IFN-γ. However, whilst HIV+ subjects from KPS had the lowest responses (and CD4 counts), HIV negative subjects from KPS also had very low responses across the board. Additionally, subjects from SUN showed no difference in CD4 levels between HIV+LTBI and HIV+TB but were the only site to show a difference in responses to PPD with active cases significantly lower than LTBI regardless of HIV status. Even after adjusting for CD4 counts, there was still considerable variation between sites in regards to antigen reactivity suggesting that other confounding factors need to be considered such as ethnicity (host genetics), nutritional status and microbial environment.

Although accuracy of combined analyses from all sites were impeded by considerable variations in responses, the main purpose of this study was the search for antigens which distinguish between TST^-^, TST^+^ (LTBI) subjects and TB patients regardless of geographical location (and hence of ethnicity, nutritional status, microbial environment, etc.). When results from all sites were combined, HIV^-^ patients with active TB showed significantly lower responses compared to both TST^-^ and TST^+^ (LTBI) contacts to latency antigens (Rv0569, Rv1733, Rv1735, Rv1737) and the rpf Rv0867; whilst responses to EC, PPD, Rv2029, TB10.3, TB10.4 and PHA were significantly higher in TST^+^ HCC (LTBI) compared to active TB and TST^-^ HCC. Fewer differences were seen in subjects with HIV co-infection, with response to PHA being significantly lower in subjects with active TB compared to those with LTBI and no difference with respect to any other antigen. Interestingly, PHA-induced polyclonal responses were often lower than PPD-induced responses to Mtb. This may relate to the highly activated immune system of TB cases making their T cells more susceptible to activation-induced cell death [17] with PHA-induced IFN-γ inversely correlating with disease severity [18].

Interestingly, the heat-shock protein, Rv2031c, which was recently used as a post-exposure sub-unit vaccine enteric to *M. avium* infection in cattle [19], induced low responses in our study with the only difference seen for HIV^+^ subjects from KPS (LTBI higher than TB). However, protection induced by Rv2031c in cattle was shown to be mediated by antibodies rather than T cells [19], emphasizing the importance for multi-parametric analysis of responses to novel vaccine candidates.

Since the inception of this study, dominant Mtb antigens were reported to be evolutionary hyperconserved [1,20] leading to the suggestion that immunogenicity to these antigens could benefit survival of Mtb. Disparities in responses between sites in our study despite a large cohort, and the relatively few differences seen between TB and LTBI, regardless of HIV status, suggests that new Mtb epitope discovery is required for determining the optimal candidates for development of novel vaccines [21,22]. Furthermore, underlying genetic influences are clearly playing a role since responses differed considerably between sites despite the use of the same batch of reagents for all assays and adjusting for age, sex and other confounders. The fact we saw few differences between TB and LTBI in this study may be explained partly by the use of a long-term culture assay, which will detect both effector and central memory cells. This may mean we are analysing responses to antigens which were expressed during the asymptomatic stage but which could not prevent progression to active disease in the TB cases. Additionally, we only measured IFN-γ in our study, which is required but not sufficient to protect against TB disease [23]. As such, we are currently assessing levels of cytokines other than IFN-γ in each of the culture supernatants to determine correlates of protection, which may be used for future vaccine efficacy trials.

In conclusion, we have shown the utility of performing large, multi-site studies for TB research. The IFN-γ ELISA is a cheap and useful tool for screening potential antigenicity in subjects across the spectrum of TB and HIV infection. These studies allow critical evaluation of responses to TB antigens in a large cohort of subjects with different TB and HIV status and genetic backgrounds: essential for finally elucidating what constitutes protective immunity to TB.

## Supporting Information

Table S1 **Institutional review boards for ethics approval at each of the study sites.**
(XLSX)

Table S2 **Description of Mtb antigens used in this study.** Mtb = *Mycobacterium tuberculosis*; PPD = purified protein derivative; rpf = resuscitation-promoting factor.
(DOCX)
