# Supplementary material for: Analysis of Host Responses to Mycobacterium tuberculosis Antigens in a Multi-Site Study of Subjects with Different TB and HIV Infection States in Sub-Saharan Africa
Source: PLoS One. 2013 Sep 10;8(9):e74080. doi: 10.1371/journal.pone.0074080 (PMC3769366; doi:10.1371/journal.pone.0074080)
Supplement: Table S2 — Description of Mtb antigens used in this study. Mtb = Mycobacterium tuberculosis; PPD = purified protein derivative; rpf = resuscitation-promoting factor. (DOCX) [file pone.0074080.s002.docx]

| Mtb stage | Accession number | Description | Mtb gene region | Protein length (aa) |
| --- | --- | --- | --- | --- |
|  | PPD | Crude Mtb antigen mix |  |  |
| Secreted | ESAT6/CFP10 | ESAT-6 and CFP-10 fusion protein |  |  |
| Dormancy | Rv0081 | Probable transcriptional regulatory protein | - | 114 |
| Secreted | Rv0288 | Low molecular weight protein antigen 7 esxH (10kd antigen) (CFP-7, TB10.4) | esxH | 96 |
| Dormancy | Rv0569 | Conserved hypothetical protein | - | 88 |
| Rpf | Rv0867c | Possible conserved trans-membrane protein (tryglycosylase, rpfA) | rpfA | 407 |
| rpf | Rv1009 | Probable rpfB (transglycosylase, C5 adhesion domain) | rpfB | 362 |
| Reactivation | Rv1131 | Cytrate synthase 3 | gltA1 | 393 |
| Reactivation | Rv1471 | Thioredoxin reductase | trxB | 123 |
| Dormancy | Rv1733c | Probable conserved transmembrane protein |  | 210 |
| Dormancy | Rv1735c | Hypothetical membrane protein |  | 165 |
| Dormancy | Rv1737c | Possible nitrite/nitrate transporter | narK2 | 395 |
| Secreted | Rv1886c | Secreted antigen 85-B FBPB (mycolyl-transferase 85B) | fbpB | 325 |
| Dormancy | Rv2028c | Conserved hypothetical protein - USPA |  | 279 |
| Dormancy | Rv2029c | Probable phosphofructokinase (pfkB) | pfkB | 339 |
| Dormancy | Rv2031c | Heat shock protein HSPX (alpha crystalline homolog) 16kd antigen Hsp16.3 | acr | 144 |
| rpf | Rv2450c | Probable resuscitation-promoting factor rpfE (transglycosylase) | rpfE | 172 |
| Starvation | Rv2659c | Probable phiRv2 prophage integrase (pool) | - | 375 |
| Starvation | Rv2660c |  |  |  |
| Secreted | Rv3019c | Secreted ESAT-6 (ESXR-TB10.3) | esxR | 96 |
| Dormancy | Rv3131 | Hypothetical protein (possible nitroreductase NfnB) | bfnB | 332 |
| Reactivation | Rv3407 | Conserved hypothetical protein | - | 99 |
